# Respiratory health effects and exposure to superabsorbent polymer and paper dust - an epidemiological study

**DOI:** 10.1186/1471-2458-11-557

**Published:** 2011-07-13

**Authors:** Mathias Holm, Anna Dahlman-Höglund, Kjell Torén

**Affiliations:** 1 All authors are from Section of Occupational and Environmental Medicine, Sahlgrenska Academy, University of Gothenburg, Sweden

## Abstract

**Background:**

The primary aim of the present study was to investigate if exposure to dust from absorbent hygiene products containing superabsorbent polymer is related to symptoms from the airways and from the eyes. The secondary aim was to estimate the current exposure to superabsorbent polymer among production and maintenance workers in a plant producing hygiene products.

**Methods:**

The cohort comprised 1043 workers of whom 689 were exposed to super absorbent polymer and 804 were exposed to paper dust (overlapping groups). There was 186 workers not exposed to either superabsorbent polymer or to paper dust They were investigated with a comprehensive questionnaire about exposure, asthma, rhinitis and symptoms from eyes and airways. The results were analyzed with logistic regression models adjusting for sex, age, atopy and smoking habits. An aerosol sampler equipped with a polytetrafluoroethylene filter with 1 μm pore size was used for personal samplings in order to measure inhalable dust and superabsorbent polymer.

**Results:**

The prevalence of nasal crusts (OR 1.4, 95% CI 1.01-2.0) and nose-bleeding (OR 1.7, 95% CI 1.2-2.4) was increased among the paper dust exposed workers (adjusted for superabsorbent polymer exposure). There were no significant effects associated with exposure to superabsorbent polymer (adjusted for paper dust exposure). The average exposure to inhalable levels of total dust (paper dust) varied between 0.40 and 1.37 mg/m^3^. For superabsorbent polymer dust the average exposure varied between 0.02 and 0.81 mg/m^3^.

**Conclusions:**

In conclusion, our study shows that workers manufacturing diapers in the hygiene industry have an increased prevalence of symptoms from the nose, especially nose-bleeding. There was no relation between exposure to superabsorbent polymer and symptoms from eyes, nose or respiratory tract, but exposure to paper dust was associated with nose-bleeding and nasal crusts. This group of workers had also a considerable exposure to superabsorbent polymer dust.

## Background

In manufacturing of hygiene products like diapers and tampons, superabsorbent polymer is used. This is a polymerized acrylate that is added to cellulose, resulting in a product with enormous ability to absorb water, which has caused an increased use of this substance in the production of hygiene products. The health effects and occupational exposure to this substance are largely unknown. In a criteria document from *Deutsche Forschungsgemeinschaft *animal studies, most of them not published in common peer-reviewed journals, are presented where inflammatory reactions in the lungs are shown after exposure to superabsorbent polymer [1]. In humans, there are no epidemiological studies published in common peer-reviewed journals on the effect of superabsorbent polymer exposure on the airways. There is also a lack of published exposure levels of this substance which seems to have irritating properties.

Workers manufacturing hygiene products are also exposed to paper dust. There are several studies where an increased prevalence of lower airways symptoms and impaired lung function has been related to occupational exposure to soft paper dust [2-5]. In addition, subjects exposed to paper dust have been shown to have an increased prevalence of different nasal symptoms [6].

The primary aim of the present study was to investigate if exposure to dust from absorbent hygiene products containing superabsorbent polymer is related to symptoms from the eyes and from the airways. The secondary aim was to estimate the current exposure to superabsorbent polymer among production and maintenance workers in a factory producing hygiene products.

## Methods

The study was performed at two Swedish factories, one production plant and one development plant. They produced hygiene products (mostly diapers and tampons) made of soft tissue paper (cellulose). Superabsorbent polymer was added to the products in order to increase the absorbing properties. At production units there are continuous working machines but at units for development the machines are started just for short periods of time and there could be long intervals between each start.

The study population comprised all workers (n = 1990) employed for more than six months 1980 to 2003. They were identified using the personnel files and deceased and emigrated workers were not included. The study population comprised production workers, supervisors and other white-collar workers. A questionnaire was mailed to all workers, and after two reminders it was completed by 1043 workers (52%). The questionnaire comprised items about occupational history, symptoms from eyes and airways, diseases, medication and smoking habits. On the basis of the questionnaire, exposure to superabsorbent polymer was defined as a positive answer to "During which years have you in your work been in exposed to superabsorbent (SAP)?" and exposure to paper dust was defined as a positive answer to "During which years have you in your work been exposed to paper dust/cellulose?". Each subject was classified as either exposed to superabsorbent polymer or not as well as either exposed to paper dust or not. These categories were partly overlapping. There were 186 subjects not reporting exposure to either superabsorbent polymer or to paper dust, and those were classified as unexposed controls.

Current asthma was defined as an affirmative answer to; "Have you during the past 12 months experienced asthmatic symptoms, i.e. periodical or attacks of difficulties to breath or dyspnoea? The symptoms can appear with or without cough and with or without wheezing".

"Physician diagnosed adult-onset asthma" was defined as a positive answer to "Have you ever had asthma diagnosed by a doctor?" and that they declared that they were more than 15 years old at diagnosis answering the next question "How old were you then?" [7,8].

Rhinitis was defined as a positive answer to "Have you after 15 years of age ever had symptoms from your nose like nasal congestion, running nose and/or sneezing attacks without having a cold?" [9].

Eye symptoms (conjunctivitis) were defined as a positive answer to "Do you have eye problems (red, running or itching eyes)?"

Nose-bleeding was defined as a positive answer to "Have you after 15 years of age ever had nose-bleeding?"

Nasal blockage was defined as a positive answer to "Have you after 15 years of age ever had nasal blockage more than three days in a row?"

Nasal crusts were defined as a positive answer to "Have you after 15 years of age had nasal crusts more than three days in a row?".

Hand eczema was defined as a positive answer to "Have you ever had hand eczema?".

Atopy was defined as "Did you had any form of allergy as a child, for instance atopic dermatitis, asthma or allergic rhinitis?".

Smoking has been defined as daily smoking during at least one year. According to their status at follow-up, smokers have been divided into "current", "former" and "never-smokers".

The items were obtained from previously used questionnaires [4-10]. These items were combined to a questionnaire. There was no separate validation for this new questionnaire, however the outcome items have been used in other questionnaires [11,12].

### Measurement of exposure

The Institute of Occupational Medicine, IOM sampler equipped with a polytetrafluoroethylene filter with 1 μm pore size was used for personal samplings in order to measure inhalable dust and super absorbent polymer [13]. The samplings were performed in the breathing zone. The airflow was 2 L/minute and the samplings were carried out at the production plant as well as at the development plant. The measurements were performed over full shifts (for practical reasons the sampling time was allowed to vary between 320 and 490 minutes). Totally, 60 samplings were performed at the unit for production and 37 at the unit for development. All working moments and machines are represented. The samplings at the unit for development represent a situation where the machines were used. The IOM sampling device follows the international convention for inhalable dust, European Standard EN 481, as previously described [14,15]. The use of personal protection devices were not considered in the measurements, however, the use of personal protection devices was uncommon.

After sampling, filters within 12 hours were transported to lab for conditioning and weighing. Gravimetric decision of amount of inhalable dust was used. The filters were then sent to AlessaChemi GmbH Frankfurt am Main for further analysis regarding super absorbent polymer.

### Statistical analyses

Statistical analyses were performed using the statistical package SAS, version 8. The outcomes are given as prevalences and the univariate analyses are based on the Mantel-Haenszel comparing exposed subjects and unexposed subjects. In addition, for the outcomes physician-diagnosed asthma, current asthma, rhinitis, nasal blockage, nose-bleeding, conjunctivitis and hand eczema the odds ratios for exposure to superabsorbent polymer and paper dust were analysed using multiple logistic regression models for each outcome with adjustments for sex, smoking habits, age, and atopy. All models comprised both exposure variables (superabsorbent polymer and paper dust) and the same adjustment variables. Smoking was handled using two dummy variables (never-smoker and ex-smoker vs. current smoking). We also analyzed interaction by stratifying for superabsorbent polymer and paper dust, respectively.

## Results

At the production plants the average exposure during 8 h for the machine operators to inhalable dust (mainly paper dust) was 1.37 mg/m^3 ^and to superabsorbent polymer it was 0.02 mg/m^3^. For the mechanics at the production plant average exposure to inhalable dust and superabsorbent polymer was 1.64 mg/m^3 ^and 0.81 mg/m^3^, respectively. At the plant for development, a mean concentration of 0.4 mg/m^3 ^for inhalable dust and 0.03 mg/m^3 ^for superabsorbent polymer was found when the machines were used. See Table 1 for more detailed information.

**Table 1:** Concentrations of inhalable dust and super absorbent polymer (SAP) among workers in two plants producing hygiene products.

Occupations or department	N	Arith-metic Mean	Range	Geometric Mean	Geometric standard deviation	95% confidence intervals
**Machine operators**						
Inhalable dust, mg/m^3^	41	1.37	0.17-23.9	0.53	2.66	0.07-3.5
SAP, mg/m^3^	41	0.02	0.002-0.23	0.01	2.75	0.002-0.09

**Mechanics**						
Inhalable dust, mg/m^3^	19	1.64	0.14-15.4	0.47	2.29	0.09-2.39
SAP, mg/m^3^	19	0.81	0.002-10.3	0.03	2.67	0.005-0.24

**Development department**						
Inhalable dust, mg/m^3^	37	0.40	0.05-1.51	0.27	2.52	0.04-1.65
SAP, mg/m^3^	37	0.03	0.002-0.40	0.01	3.45	0.008-0.11

Basic data of the population is listed in Table 2 and Table 3. The majority of the subjects (n = 636) were exposed to both superabsorbent polymer and paper dust, 53 reported only exposure to superabsorbent polymer and 168 subjects reported exposure only to paper dust. There were 186 subjects that were classified as unexposed to both superabsorbent polymer and paper dust, thus serving as the control group. The prevalence of asthma, nasal symptoms, conjunctivitis and hand eczema in the different exposure groups are shown in Table 2 and Table 3.

**Table 2:** Basic data about the population, separated into all subjects and the overlapping groups of workers exposed to super absorbent polymer (SAP) or paper dust.

	All	Exposed to superabsorbent polymer (SAP)^1^	Exposed to paper dust^1^
All	1043	689	804

Men	589 (56.5%)	414 (60.1%)	472 (58.7%)

Women	454 (46.5%)	275 (39.9%)	332 (41.3%)

Age, yrs	44.8 (11.3)	42.7 (10.4)	44.0 (10.8)

Never smokers	523 (50.1%)	361 (52.4%)	405 (50.4%)

Ex-smokers	330 (31.7%)	207 (30.0%)	254 (31.6%)

Current smokers	190 (18.2%)	121 (17.6%)	145 (18.0%)

Atopy	197 (18.9%)	148 (21.5%)	160 (19.9%)

Physician-diagnosed adult-onset asthma	71 (6.8%)	40 (5.8%)	50 (6.2%)

Current asthma	112 (10.7%)	72 (10.5%)	84 (10.5%)

Rhinitis	471 (45.2%)	322 (46.7%)	378 (47.0%)^3^

Nasal blockage	349 (33.5%)	252 (36.6%)^2^	283 (35.2%)^3^

Nasal crusts	442 (42.4%)	313 (45.4%)^2^	362 (45.0%)^2^

Nose-bleeding	465 (44.6%)	332 (48.2%)^2^	387 (48.1%)^2^

Conjunctivitis	235 (22.3%)	162 (23.5%)	188 (23.4%)

Hand eczema	134 (12.9%)	89 (12.9%)	104 (12.9%)

1. Overlapping groups

2. p < 0.01

3. p < 0.05

**Table 3:** Basic data about the population, separated into unexposed subjects, subjects exposed either to super absorbent polymer or paper dust and subjects with both exposures.

	No exposure to superabsorbent polymer or paper dust	Only exposed to superabsorbent polymer	Only exposed to paper dust	Exposed to both superabsorbent polymer and paper dust
All	186	53	168	636

Physician-diagnosed adult-onset asthma	17 (9.1%)	4 (7.6%)	14 (8.3%)	36 (5.7%)^2^

Current asthma	20 (10.8%)	8 (15.1%)	20 (11.9%)	64 (10.1%)

Rhinitis	71 (38.2%)	22 (41.5%)	78 (46.4%)	300 (47.2%)^3^

Nasal blockage	46 (24.7%)	20 (37.7%)^1^	51 (30.4%)	232 (36.5%)^3^

Nasal crusts	61 (32.8%)	19 (35.9%)	68 (40.5%)	294 (46.2%)^3^

Nose-bleeding	62 (33.3%)	16 (30.2%)	71 (42.3%)^2^	316 (49.7%)^3^

Conjunctivitis	35 (18.8%)	12 (22.6%)	38 (22.6%)	150 (23.6%)

Hand eczema	22 (11.8%)	8 (15.1%)	23 (13.7%)	81 (12.7%)

P-values in comparison with unexposed subjects.

1. p = 0.06

2. p = 0.08

3. p < 0.05

In the univariate analyses, without taking into account overlapping exposures, exposure to paper dust vs. no exposure to paper dust was associated with an increased prevalence of rhinitis and nasal symptoms such as nasal blockage and nasal crusts (Table 2). Exposure to superabsorbent polymer vs. no exposure to superabsorbent polymer was associated with an increased prevalence of nasal blockage and nasal crusts. Nose-bleeding was associated both with exposure to superabsorbent polymer or to paper dust. When performing univariate analysis in the separate exposure groups (Table 3), the combined exposure to superabsorbent polymer and paper dust was associated with different nasal symptoms, including nose-bleeding. Exposure to superabsorbent polymer (only) was associated with nasal blockage and exposure to paper dust (only) was associated with nose-bleeding.

As the exposure categories were overlapping, an analysis with multiple logistic regression models comprising both exposure to SAP and paper dust was performed (Table 4). These models showed an increased odds ratio for nasal crusts (OR 1.4, 95% CI 1.01-2.1) and nose-bleeding (OR 1.7, 95% CI 1.2-2.4) associated with paper dust exposure. We also analysed the interaction between exposure to paper dust and SAP by stratifying the models. Among workers exposed to SAP, exposure to paper dust was significantly associated with nose-bleeding (OR 2.2, 95% CI 1.2-4.1). No other interactions were found (data not shown).

**Table 4:** Odds ratios for asthma, current asthma, rhinitis, different nasal symptoms, conjunctivitis and hand eczema in relation to exposure to super absorbent polymer and paper dust.

	Exposure to SAP		Exposure to paper dust	
Outcome	OR	95% CI	OR	95% CI

Physician-diagnosed adult-onset asthma (n = 71)	0.8	0.4-1.4	0.8	0.5-1.6

Current asthma (n = 112)	1.0	0.6-1.7	0.9	0.5-1.5

Rhinitis (n = 471)	0.9	0.7-1.3	1.3	0.9-1.9

Nasal blockage (n = 349)	1.3	0.9-1.8	1.2	0.8-1.7

Nasal crusts (n = 442)	1.1	0.8-1.5	1.4	1.01-2.1

Nose bleeding (n = 465)	1.1	0.8-1.5	1.7	1.2-2.4

Conjunctivitis (n = 235)	1.1	0.7-1.6	1.2	0.8-1.8

Hand eczema (n = 134)	0.9	0.6-1.5	1.0	0.6-1.7

The results are from logistic regression models comprising gender, atopy, smoking, age and both exposure variables. OR = odds ratio. CI = confidence interval.

1. 1 = Yes; 0 = No

## Discussion

The most important finding from this study, based on logistic regression modelling, was that exposure to paper dust increased the odds ratio for nose-bleeding and nasal crusts. It was also shown that operators at plants producing hygiene products had a low, but measurable, exposure to superabsorbent polymer, ranging from 0.02 mg/m^3 ^to 0.81 mg/m^3^.

The strength of the present study is the size of the sample which made it feasible to investigate effects of exposure to paper dust and superabsorbent polymer and also to perform proper adjustments for confounders. One weakness is the overlapping exposures causing a loss of power. The measured exposure levels of superabsorbent polymer are adding new knowledge as such data is lacking in the literature.

A main weakness of the study is the healthy-worker bias that is clearly operating in this study population, as shown by the significantly lower asthma prevalence among the dust exposed workers. This may indicate that we may underestimate exposure related differences in this group of workers. However, our intention was to minimize this bias by including all workers who after 1990 has worked more than 6 months, comprising also workers who have left the factories.

The response rate in the present study was not high, 52 percent. This may affect the prevalence of different outcomes, but it has been shown that low response rates will only marginally affect the odds ratios [16,17].

The main component of the produced hygiene products was cellulose and it was therefore assumed that the inhalable dust mainly consisted of paper dust (cellulose). However, in a few samples superabsorbent polymer was the main component of the inhalable dust. It should be noted that superabsorbent polymer was detected in all samples made, which implies that it is spread all over the working area. The concentrations of superabsorbent polymer that we measured seem to be in the same range as previously reported [1]. The estimated concentrations of cellulose dust in the present study are generally low compared to those found in a soft-paper mill [2]. However, there were some very high values among mechanics in the supporting staff, which could be explained by their tasks.

There was a markedly increased odds ratio for nose-bleeding among the paper dust exposed workers (OR 1.7, 95% CI 1.2-2.4). In addition, there was an increased odds ratio for nasal crusts in relation to paper dust exposure. Exposure to paper dust probably causes a drying effect of the nasal mucosa which, in turn, increases the susceptibility of the mucosa. This may explain the increased occurrence of nose-bleeding, a more severe symptom than nasal crusts and blockage. Even if we did not find any effect of exposure to superabsorbent polymer the increased occurrence of nose-bleeding may be a joint effect of exposure to superabsorbent polymer and to paper dust. The interaction analysis supported to some extent such an assumption.

An increased prevalence of nose-bleeding has previously not been reported among paper workers, but in workers exposed to diisocyanates and phtalic anhydrides [18,19]. An increased occurrence of nasal crusts has previously been observed among workers exposed to soft paper dust [6,20]. In a previous Swedish cross-sectional study it was found that exposure to inhalable paper dust in concentrations above (median level 3.6 mg/m^3^, inhalable dust) those found in the present study was associated with an increased prevalence of nasal blockage and nasal crusts without any objective signs of inflammation in the nasal mucosa [6].

The lack of effects in the present study of both paper dust and super absorbent polymer dust regarding the lower airways is interesting. Studies have reported impaired lung function and an increased prevalence of symptoms from the lower airways among paper mill workers exposed to high levels of soft paper dust (more than 5 mg/m^3^, total dust) [2,3,20]. However, in our study the workers probably were exposed to concentrations of paper dust too low to induce symptoms from the lower respiratory tract.

In humans, there are no epidemiological studies on the effect of superabsorbent polymer exposure on the airways published in common peer-reviewed journals. In the criteria document from *Deutsche Forschungsgemeinschaft *it is referred to a study where super absorbent polymer exposure levels below 0.01 mg/m^3 ^respirable dust do not show any long term effects on lung function in humans [1]. It is also referred to cross-sectional studies from NIOSH where an increased prevalence of unspecific irritation of the eyes, nose and respiratory tract was indicated in groups exposed to approximately 0.1 mg/m^3 ^superabsorbent polymer (total dust), but no effects regarding changes in lung function [1]. There are animal studies showing inflammatory reactions in the lungs at concentrations around 0.3 - 1.0 mg/m^3 ^[21,22], levels that have been found in the present plants. However, in the present study there was a lack of associations between exposure to superabsorbent polymer and symptoms from the eyes, nose and lower respiratory tract. Perhaps the concentrations were too low to induce symptoms and thus, certainly, too low to start inflammatory reactions.

## Conclusions

Our study shows that workers manufacturing diapers in the hygiene industry have an increased prevalence of symptoms from the nose, especially nose-bleeding. There was no relation between exposure to superabsorbent polymer and symptoms from eyes, nose or respiratory tract, but exposure to paper dust was associated with nose-bleeding and nasal crusts. This group of workers had also a considerable exposure to superabsorbent polymer dust.

## Competing interests

The authors declare that they have no competing interests.

## Authors' contributions

MH carried out the statistical analysis and drafted the first version of the manuscript. ADH carried out the exposure measurements. KT designed the study and participated in the statistical analyses. All authors read and approved the final manuscript.

## Pre-publication history

The pre-publication history for this paper can be accessed here:

http://www.biomedcentral.com/1471-2458/11/557/prepub
